# Drought tolerance of the photosynthetic apparatus of bread wheat (Triticum aestivum L.) lines with introgressions in chromosome 2D from Aegilops tauschii Coss.

**DOI:** 10.18699/vjgb-25-56

**Published:** 2025-07

**Authors:** S.V. Osipova, A.V. Permyakov, A.V. Rudikovskii, E.G. Rudikovskaya, T.A. Pshenichnikova

**Affiliations:** Siberian Institute of Plant Physiology and Biochemistry of the Siberian Branch of the Russian Academy of Sciences, Irkutsk, Russia Irkutsk State University, Irkutsk, Russia; Siberian Institute of Plant Physiology and Biochemistry of the Siberian Branch of the Russian Academy of Sciences, Irkutsk, Russia; Siberian Institute of Plant Physiology and Biochemistry of the Siberian Branch of the Russian Academy of Sciences, Irkutsk, Russia; Siberian Institute of Plant Physiology and Biochemistry of the Siberian Branch of the Russian Academy of Sciences, Irkutsk, Russia; Institute of Cytology and Genetics of the Siberian Branch of the Russian Academy of Sciences, Novosibirsk, Russia

**Keywords:** bread wheat, soil drought, shoot biomass, gas exchange, chlorophyll fluorescence, introgressions, molecular markers, мягкая пшеница, почвенная засуха, биомасса побега, газообмен, флуоресценция хлорофилла, интрогрессии, молекулярные маркеры

## Abstract

One of the ways to increase yield stability of bread wheat under changing climatic conditions is through improving the photosynthesis efficiency. For this purpose, various genetic strategies are used. They include marker-assisted selection and the use of the genetic potential of wild wheat relatives. Previously, using introgression wheat lines carrying different segments of chromosome 2D from Aegilops tauschii in the genetic background of the wheat (Triticum aestivum) variety Chinese Spring (CS), we mapped QTLs associated with variability in shoot biomass and gas exchange under contrasting water supply conditions. In this work, by “splitting” the primary introgressions, we obtained secondary introgression CS lines with reduced segments of Ae. tauschii introgressions in the short and long arms of chromosomes 2D. The aim of this study was to investigate the tolerance of the photosynthetic apparatus to soil water deficit in these lines. We estimated the size of drought effect on shoot biomass, gas exchange parameters, photosynthetic pigment content, slow and fast chlorophyll fluorescence parameters, and fast light curve parameters. The results showed that line 1004 with an introgression in chromosome 2DS limited by microsatellite loci Xgwm296 and Xgwm261 was little affected by drought in respect of the chlorophyll (a+b)/carotenoid ratio and primary photosynthetic processes. In line 1005 with a single introgression in the region of the Xgwm261 marker, the chlorophyll (a+b)/carotenoid ratio and indicators of the functional activity of photosystems significantly decreased under water deficiency. The chlorophyll (a+b)/carotenoid ratio, CO2 assimilation rate, and chlorophyll fluorescence parameters remained stable in line 1034 with an introgression in chromosome 2DL near the Xgwm1419 and Xgwm157 loci. In line 1021 with an introgression in the region of the Xgwm539 marker on the same chromosome, we observed a strong negative effect of drought on the rate of CO2 assimilation and indicators of the functional activity of photosystems. The Xgwm1419 and Xgwm296 markers can be recommended for use in marker-assisted breeding for drought tolerance of bread wheat in the cases where Ae. tauschii acts as a donor of genetic material.

## Abstract (ru)

Улучшение эффективности фотосинтеза в изменяющихся климатических условиях является одним из способов повышения стабильности урожая сельскохозяйственных растений. Для этого применяют различные генетические стратегии, в частности маркер-ориентированную селекцию, а также привлекают генетический потенциал диких сородичей пшеницы. Ранее, используя интрогрессивные линии пшеницы, содержащие различные сегменты хромосомы 2D от Aegilops tauschii в генетическом фоне пшеницы Triticum aestivum сорта Чайниз Спринг (ЧС), мы картировали QTL, ассоциированные с вариабельностью биомассы побега и газообмена в контрастных условиях водоснабжения. В данной работе путем «дробления» первичных интрогрессий мы получили вторичные интрогрессивные линии пшеницы ЧС с более короткими сегментами интрогрессий от Ae. tauschii. Целью исследования было изучить устойчивость фотосинтетического аппарата к дефициту воды в почве у вторичных интрогрессивных линий, содержащих редуцированные интрогрессии от Ae. tauschii в коротком и длинном плечах хромосомы 2D. Мы оценили размер эффекта засухи на биомассу побега, параметры газообмена, содержание фотосинтетических пигментов, параметры медленной и быстрой флуоресценции хлорофилла и параметры быстрых световых кривых. Результаты показали, что у линии 1004 с участком интрогрессии в хромосоме 2DS, ограниченном микросателлитными локусами Xgwm296 и Xgwm261, засуха незначительно влияла на соотношение хлорофиллы a+b/каротиноиды и первич- ные процессы фотосинтеза. У линии 1005 с участком интрогрессии в районе маркера Xgwm261 при дефиците воды значительно снижались соотношение хлорофиллы a+b/каротиноиды и показатели функциональной активности фотосистем. У линии 1034 с интрогрессией в хромосоме 2DL в районе локусов Xgwm1419 и Xgwm157 соотношение хлорофиллы a+b/каротиноиды, скорость ассимиляции СО2 и параметры флуоресценции хлорофилла при засухе оставались стабильными. У линии 1021 с участком интрогрессии в районе маркера Xgwm539 на этой же хромосоме мы наблюдали сильное негативное влияние засухи на скорость ассимиляции СО2 и показатели функциональной активности фотосистем. Маркеры Xgwm1419 и Xgwm296 можно рекомендовать для использования в маркер-ориентированной селекции на засухоустойчивость мягкой пшеницы в случаях, когда донором генетического материала выступает Ae. tauschii.

## Introduction

Improving the efficiency of photosynthesis is considered one of the most important issues of breeding work aimed at increasing productivity of bread wheat (Triticum aestivum L.) through improving tolerance to unfavorable factors. Various genetic strategies are effective in achieving these goals, including the use of the genetic potential of wild relatives of wheat and marker-assisted selection (Reynolds et al., 2012).

Wild relatives represent a valuable gene pool for bread wheat improvement, since this crop has a limited genetic diversity for meeting the challenges of modern breeding. Various species of the genus Aegilops L., which is most closely related to the genus Triticum L., are considered a source of beneficial alleles for bread wheat fortification against abiotic stresses, pests, and diseases (Przewieslik-Allen et al., 2019; Pour- Aboughadareh et al., 2021). One such species is Ae. tauschii, known as the donor of the D genome of bread wheat and containing favorable allelic variations in genes associated with stress responses (Jia et al., 2013). Its homology with the D subgenome of bread wheat simplifies the introgression process during breeding and for genetic analysis. Therefore, Ae. tauschii is widely used in research aimed at improving the productivity and stability of wheat under various climatic conditions (Nyine et al., 2021; Ma et al., 2023).

An intermediate step in the transfer of genetic diversity from this genome is synthetic hexaploid wheats with the BBAADD genome, homologous to bread wheat. The first synthetic, called Synthetic 6x (Syn6x) (McFadden, Sears, 1946), was used to obtain single-chromosome substituted lines of Chinese Spring (CS)(Syn6x) (Nicholson et al., 1993). Subsequently, on the basis of substitution lines D-genome chromosomes, introgressive lines carrying single chromosome segments from Ae. tauschii of different sizes were obtained (Pestsova et al., 2001). Using this set of eighty introgressive lines CS(Syn6x), we mapped quantitative trait loci (major QTL) associated with variability in shoot biomass (SB) and gas exchange parameters under soil water deficit in two regions of chromosome 2D (Osipova et al., 2016). One of the regions was located on the short arm between microsatellite markers Xgdm5 and Xgwm296, and the second was flanked by markers Xgwm539 and Xgwm1419 on the long arm. The size of the first region was 11.4 cM, and the second, 10.5 cM (Röder et al., 1998).

Further refinement of the position of loci associated with photosynthesis variability on chromosome 2D and search for putative candidate genes became possible by obtaining the lines with reduced segments of introgressions and studying the stability of the functioning of the photosynthetic apparatus in the new lines. Chlorophyll (Chl) fluorescence parameters are considered a reliable source of information about the physiological state of photosynthetic apparatus of plants (Goltsev et al., 2016). They have been successfully used in screening of adult bread wheat plants for drought tolerance in the field and in that of wheat seedlings in the laboratory (Botyanszka et al., 2020; Peršić et al., 2022).

The aim of this study was to investigate the tolerance of the photosynthetic apparatus to soil water deficiency in the introgressive lines containing short introgressions from Ae. tauschii.

## Materials and methods

Genetic material, molecular analysis and experimental conditions. The two groups of secondary introgressive lines (SILs) obtained on the basis of two introgressive lines, Chinese Spring CS(Syn6x 2D-4) and CS(Syn6x 2D-6) (Pestsova et al., 2001), along with a recipient variety CS were used in the work. To narrow down the regions of introgressions, a “splitting” approach was used, consisting of hybridization of these two primary lines with the recipient CS. Secondary introgressive lines were obtained by a single backcrossing followed by subsequent self-pollination into F2. The plants were then analyzed for their microsatellite marker composition. Those plants were selected that showed allelic differences in the target chromosome 2D regions where clusters of QTL loci associated with drought response had previously been detected (Osipova et al., 2016).

**Fig. 1.**
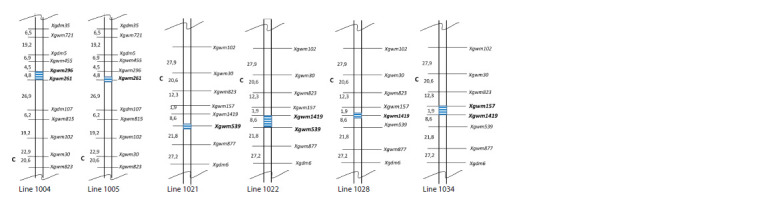
Schematic arrangement of introgression regions of chromosome 2D in secondary introgressive lines CS(Syn6x 2D-4) (lines 1004 and 1005) and CS(Syn6x 2D-6) (lines 1021, 1022, 1028 and 1034). Microsatellite markers of chromosome 2D represented by allelic variants of Syn6x and relevant to the previously identified positions of QTL clusters associated with drought response (Osipova et al., 2016) are shown in bold. The sequence of markers and the distances between them (not to scale) are presented according to the maps of M.S. Röder et al. (1998) and E.G. Pestsova et al. (2001).


DNA extraction was performed according to the protocol of Plaschke et al. (1995). The obtained PCR products were separated in 3 % agarose gel and photographed under UV light using the Molecular Imager® Gel DocTM XR+ system (Bio- Rad Laboratories, Inc., California, USA). Two lines, numbered 1004 and 1005, were selected among F2 plants from the cross between CS and IL CS(Syn6x 2D-4) (Fig. 1). These lines differed only in the allelic state of marker Xgwm296. Allelic variants of this marker obtained using the PCR reaction are presented in Figure S1 in Supplementary Material1.

Supplementary Materials are available in the online version of the paper: https://vavilov.elpub.ru/jour/manager/files/Suppl_Osipova_Engl.xlsx


Four lines numbered 1021, 1022, 1028 and 1034 were selected among F2 plants from the cross between CS and IL CS(Syn6x 2D-6). They differ in the allelic state of markers Xgwm1419, Xgwm157 and Xgwm539 (Fig. S2). The plants were grown under controlled conditions in a CLF PlantMaster climate chamber (CLF Plant Climatic GMBH, Germany) installed in the phytotron of SIPPB SB RAS, with a 16-hour photoperiod, a temperature of 23 °C during the day and 16 °C at night, air humidity of 60 % and a light intensity of 300 μmol/(m2·s). Ten grains of each genotype were sown in two Mitscherlich pots filled with a mixture of humus, sand and peat (1:1:1). The moisture content of the soil in one pot was maintained at an optimal level (60 % of the total soil moisture capacity). In the second pot, watering was reduced by half, to 30 % of the total soil moisture capacity starting from the third leaf stage. The water regime was maintained gravimetrically. At the flowering stage, the gas exchange parameters and chlorophyll (Chl) fluorescence were measured in plants. At this stage, the main shoot mass was measured and samples were collected to determine the content of photosynthetic pigments.

Gas exchange, chlorophyll fluorescence and photosynthetic pigment content. Net photosynthesis rate (A), stomatal conductance (Gs) and transpiration rate (E) were measured using a portable leaf gas exchange system GFS-3000 (Heinz Walz, Germany). The following values of light intensity, CO2 concentration, relative humidity, temperature and airflow rate were set: 800 μmol/(m2·s), 400 μmol/mol, 60 %, 25 °C and 750 μmol/s, respectively. Water use efficiency (WUE) was calculated as A/E. The mean values and standard deviations for gas exchange parameters are given in Table S1.

Using a PAM 2500 fluorimeter (Heinz Walz, Germany) integrated with PamWin 3.05 software, the following parameters were measured: the kinetics of slow Chl fluorescence induction; the parameters of fast light curve; the kinetics of fast Chl fluorescence induction (OJIP test). To record the minimum Chl fluorescence yield in the dark-adapted state (F0), the leaves were darkened for 30 min and then illuminated with modulated measuring light of low-frequency (5 Hz) and low-intensity (630 nm). The chlorophyll fluorescence intensity under conditions of closed reaction centers (Fm) was measured after exposure to a high-intensity light pulse of 25,000 μmol (photon)/(m2·s), wavelength 630 nm, 0.1 s. Red actinic light (677 μmol photons/(m2·s)) was used to maintain photosynthesis and achieve a steady state (F). Based on the measured values of chlorophyll fluorescence parameters, the PamWin 3.50 program calculated other parameters. We assessed the response to rapid irradiance increases (every 30 s) by exposing leaves to light intensities ranging from 0 to 1,935 μmol/(m2·s) PAR photons and recorded the initial slope of the fast light response curve (α), maximum electron transfer rate (ETRmax) and minimum saturating irradiance (Ik). Chl fluorescence induced by strong light pulses was sampled in the range from 0.1 to 300 ms in the View instrument mode under the Fast Kinetics tab (Chen K. et al., 2013, Srivastava et al., 2021). All Chl fluorescence parameters measured and calculated during the study, as well as the size of drought effect (SDE) on each parameter, are listed and described in Table S2

Determination of photosynthetic pigments content. The preparation and measurement of the optical density of extracts containing photosynthetic pigments were carried out according to the previously described method (Osipova et al., 2024). To calculate the content of chlorophyll a (Chl a), chlorophyll b (Chl b) and carotenoids (Car) in the leaves, the formulas given in the work of D. Wettstein (1957) were used.

Search for coordinates of molecular markers and candidate genes that may participate in the formation of drought tolerance on chromosome 2D. To search for coordinates of markers Xgwm261 and Xgwm157, the primer sequences presented in the GrainGenes information resource (https://wheat.pw.usda.gov/GG3) were used. For marker Xgwm1419, the primer sequences were provided by Martin Ganal (TraitGenetics GmbH). Using the BLASTN program, coordinates were determined for them in the wheat genome assembly Chinese Spring IWGSC RefSeq v2. The coordinates of markers Xgwm296 and Xgwm539 are specified in this assembly (https://wheat.pw.usda.gov, last accessed February 05, 2025). In the regions limited by these markers, a search for the most probable candidate genes was carried out. Candidate genes annotated by the International Wheat Genome Sequencing Consortium (IWGS, 2018) with a high degree of reliability were considered.

Statistics. A single plant was taken as a biological replicate. At the flowering stage, gas exchange parameters were measured in six plants of each genotype under each water regime. Chl fluorescence parameters were measured in three plants of each genotype. Then, the aboveground part of the main shoot of nine plants of each genotype was cut off and weighed. Three samples taken from the flag leaves of three plants were frozen with liquid nitrogen and stored at –70 °C for subsequent determination of pigment content. The pigment content is given in mg/g fresh leaf weight. The tables present the average values ± standard deviations. The effect of soil water deficit on chlorophyll fluorescence, pigment content, and shoot biomass was assessed using the size of drought effect (SDE) index (Hedges, Olkin, 1985). The formulas for calculating SDE are given in the work of S.V. Osipova et al. (2024). The higher the effect size, the greater the increase in the parameter under drought conditions compared to the control. Negative values indicate a decrease in the parameter compared to the control.

All calculations, including average values, pooled standard deviation, adjusted SDE value, and diagram plotting were performed in Microsoft Excel, version 14.0.7268.5000 (Microsoft Corporation, 2010). The significance of differences was assessed using Student’s t-test. The fluorescence data pool was processed using nonmetric multidimensional scaling in Past, version 3.01 (Hammer et al., 2001).

## Results

**Shoot biomass and photosynthetic pigments content in leaves**

The biomass of the main shoot in the lines varied from 2.6 to 3.5 g in the control and from 1.5 to 2 g under soil drought conditions (Table 1). Under normal watering, only one line, 1005, exceeded the recipient for this trait, and the lowest value was found in line 1021. Under water deficit conditions, the shoot biomass in two lines, 1028 and 1034, exceeded the values of this trait in CS, while in lines 1021 and 1022, on the contrary, it did not reach CS values. SDE was negative in all the studied genotypes, but the value of this indicator varied from –1.69 in line 1021 to –7.06 in line 1005. Line 1021 was distinguished by reduced values of shoot biomass, both under optimal irrigation and under drought conditions. The high shoot biomass of line 1005 in the control was significantly reduced under drought. Line 1004 was more stable than line 1005 in this trait.

**Table 1.**
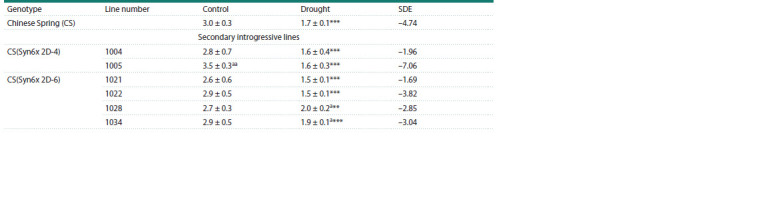
Shoot biomass (g) and its size of drought effect (SDE) of the recipient Chinese Spring and the studied lines at the flowering stage in the control and under water deficit ** p < 0.01, *** p <0.001 – significant differences between each genotype in control and drought conditions; а p < 0.05; аа p < 0.01 – significant differences between CS and lines

Under drought conditions, the content of Chl a, Chl b and Car in CS decreased, and SDE on Chl a+b/Car ratio was close to zero (Table 2). Pigment content in leaves of the lines changed differently. In lines 1004 and 1034, Chl a content significantly increased under drought. Car content also increased in all lines, except for line 1022. The greatest positive effect of drought on Car content was observed in lines 1004, 1005 and 1034, in ascending order. Chl a+b/Car ratio changed insignificantly in all genotypes, except for line 1005. The negative effect of drought on this trait in line 1005 was due to the fact that Chl a content remained stable under different conditions, and Car content increased.

**Table 2.**
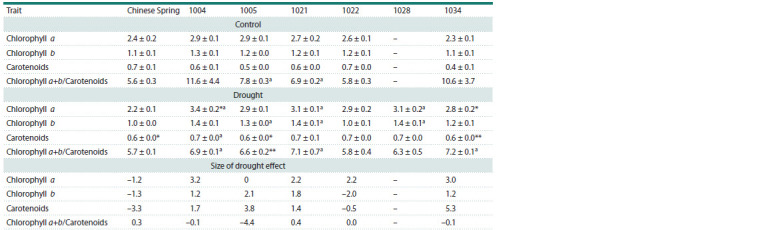
Content of photosynthetic pigments (mg/g of raw weight) and their size of drought effect in the leaves of the recipient Chinese Spring and the studied lines (n = 9) in the control and under water deficiency * p < 0.05, ** p < 0.01 – significant differences between each genotype in control and drought conditions; а p < 0.05; аа p < 0.01 – significant differences between CS and lines.

**Gas exchange and chlorophyll fluorescence**

**Fig. 2.**
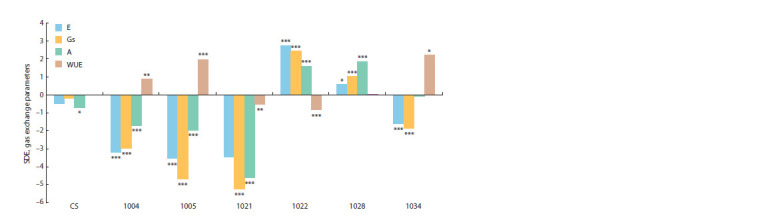
Size of drought effect (SDE) on transpiration rate (E), stomatal conductance (Gs), photosynthetic rate (A) and water use efficiency (WUE) in CS and secondary recombinant introgressive lines. * p <0.05, ** p <0.01, *** p < 0.001 – significant differences between average values of the traits in control and drought conditions.

Figure 2 shows SDE values for gas exchange parameters of CS and six secondary introgressive lines. Of all the studied genotypes, the recipient had the most stable gas exchange parameters, although its net photosynthesis rate significantly decreased under drought. The lines with introgression in the short arm of chromosome 2D showed similar changes for these traits. E and Gs significantly decreased, while net photosynthesis rate, to a lesser extent. As a result, WUE increased under drought. The lines with introgressions in the long arm of 2D chromosome showed various changes in gas exchange parameters. In line 1021, all gas exchange parameters, as well as WUE were significantly reduced. Lines 1022 and 1028 demonstrated atypical stomatal effects, increased E, Gs, and net photosynthesis rates under drought conditions. Line 1034 showed a classic adaptive response to water deficit, with decreased E and Gs, and stable net photosynthesis rate, resulting in a significant increase in WUE under drought.

To reveal the influence of introgressions on variability of Chl fluorescence parameters under water deficit conditions, we applied multidimensional nonmetric scaling of SDE indices for 39 Chl fluorescence parameters (Fig. S3). Three lines (1004, 1022, and 1028) formed a tight cluster with CS, indicating minor differences in SDE among these genotypes. This suggests that the existing introgression has a small effect on the structural and functional characteristics of photosynthetic apparatus under drought. Three other lines (1005, 1021, and 1034) were located at a significant distance from CS. This indicated significant differences in the responses of photosynthetic apparatus of these lines from others included in the cluster.

**Fig. 3.**
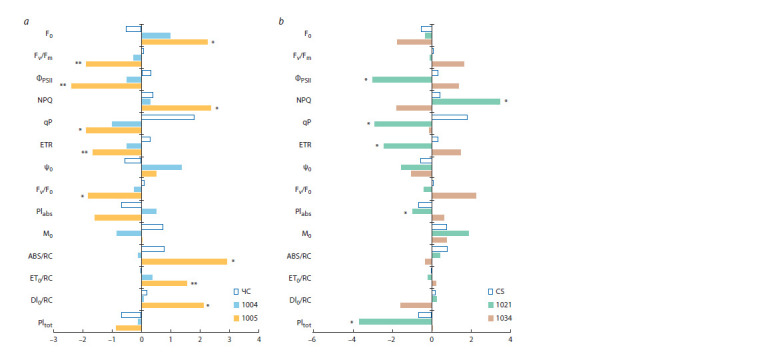
Size of drought effect for Chl fluorescence parameters in CS and secondary introgressive lines 1004 and 1005 (a); 1021 and 1034 (b). The Chl fluorescence parameters (after Goltsev et al., 2016): F0 – the minimum fluorescence of dark-adapted leaves; Fv/Fm – the maximum photochemical activity of photosystem II (PSII); ΦPSII – the effective quantum yield of PSII; NPQ – the non-photochemical fluorescence quenching; qP – the photochemical fluorescence quenching; ETR – the rate of linear electron transport through the photosystems; Ψ0 – the efficiency with which an exciton captured by a reaction center moves an electron along the chain after QA; Fv/F0 – the ratio of the rate constants of the primary photochemical reaction to the total rate of non-photochemical losses; PIIabs – an indicator of the functional activity of PSII related to the absorbed energy; M0 – a parameter that reflects the rate of closure of the reaction centers of PSII; ABS/RC – the energy flow absorbed by one reaction center; DI0/RC – the total amount of energy dissipated by one reaction center; PItot – an indicator of the functional activity of PSII, PSI and the electron transport chain between them. * p <0.05; ** p <0.01, significant differences between average values of the traits in control and drought conditions.

Next, we compared the size of drought effect on Chl fluorescence parameters in the recipient CS and lines 1004 and 1005 with introgression in the short arm of chromosome 2D (Fig. 3a) and in CS and lines 1021 and 1034 with introgressions in the long arm of the same chromosome (Fig. 3b). Figure 3 demonstrates that CS had relatively stable Chl fluorescence parameters under different irrigation conditions. The same is true for line 1004. Line 1005, on the contrary, demonstrated large differences for Chl fluorescence in the control and under drought indicating a stressed state of the photosynthetic apparatus under water deficiency. This conclusion follows from a statistically significant increase in F0 and NPQ under drought, a decrease in Fv/Fm, ΦPSII, Fv/F0 and ETR, as well as the productivity indices PIabs and PItot. Lines 1021 and 1034 with introgressions in the long arm of chromosome 2D differed significantly in their responses to drought (Fig. 3b). The photosynthetic apparatus of line 1034 adapted well to water deficit as indicated by an insignificant difference between the average values in the control and under drought and a zero value of SDE for PItot. In line 1021, ФPSII, qP, ETR and both productivity indices (PIabs and PItot) significantly decreased under drought. These changes indicated a stressed state of the photosynthetic apparatus under water deficit.

## Discussion

**Effect of introgression from Ae. tauschii in the short arm of chromosome 2D on the stability of photosynthetic processes and shoot biomass.**Line 1004 carried the introgression in chromosome 2D region flanked by markers Xgwm296 and Xgwm261, which is limited by coordinates 2D:18085000‒19623173 bp. Line 1005 carried the introgression in the region adjacent to marker Xgwm261. The lines differed substantially in the magnitude of SDE on chlorophyll and carotenoid content. This variability of photosynthetic pigments content and Chl a+b/Car ratio was more favorable for drought adaptation in line 1004 than in line 1005. In both lines, the size of PSII light-harvesting antenna increased during drought, but in line 1005, this adaptation did not result in efficient energy use. The energy flux absorbed by one PSII reaction center during drought (ABS/RC) increased in this line compared to the recipient CS, while energy dissipation (DI0/RC parameter) increased. In line 1005, F0 significantly increased under drought indicating disturbances in the excitation energy transfer in antenna and from the antenna to PSII reaction center (Goltsev et al., 2016). We observed a similar effect earlier on other wheat genetic material, in Saratovskaya 29 lines with modifications in the distal region of the short arm of chromosome 2A (Osipova et al., 2023), indicating the involvement of these chromosomal regions of the second homoeologous group in the control of primary photosynthesis processes.

The only difference between lines 1004 and 1005 was the introgression from Ae. tauschii in the region of marker Xgwm296, which was detected in line 1004 (Fig. 1). The genes associated with this marker probably determined the observed differences in drought tolerance of the two lines. The most likely candidate gene to explain them may be the gene TraesCS2D03G0092600 encoding the plant-specific transcription factor (TF) TCP. The coordinates of this gene are 2D:18667052–18667318 bp (https://wheat.pw.usda.gov/ cgi-bin/GG3). The TCP family of TFs regulate cell division, affect meristem growth (Cubas et al., 1999), and are involved in regulating responses to external signals (Danisman, 2016). The gene TraesCS2D03G0092600 was likely involved in the formation of high shoot biomass, characteristic of line 1005 under optimal watering, an advantage that the line lost under drought. The same gene is presumably associated with large differences between lines 1004 and 1005 in Chl a accumulation under water deficit, since TCP TFs have been shown to regulate chlorophyll biosynthesis in Arabidopsis (Zhen et al., 2022). In general, the physiological differences between the two lines were that line 1005 exhibited an imbalance between growth and adaptation to water stress, while line 1004 remained relatively stable.

**Effect of introgression from Ae. tauschii in the long arm of chromosome 2D on photosynthesis stability. ** We studied four lines with introgressions of different size in the region of the long arm of chromosome 2D, limited by markers Xgwm157, Xgwm1419 and Xgwm539. Line 1034 with introgression in the region of markers Xgwm1419 and Xgwm157 and line 1021 with introgression in the region of marker Xgwm539 were the most contrasting in stability. Line 1034 was distinguished by the stability of net photosynthesis and chlorophyll fluorescence indices, as well as by the relatively stable shoot biomass. In line 1021, on the contrary, the stability of photosynthetic parameters and shoot biomass were reduced compared to CS. The marker Xgwm539 coordinates are 2D:515210161–515210309 bp. This region has a very high gene density. We believe that the most likely candidate gene for explaining the negative effect of introgression in line 1021 is the gene under the number TraesCS2D03G008700 with coordinates 2D:515214093– 515217180 bp (https://wheat.pw.usda.gov/cgi-bin/GG3). One of the two transcripts of this gene is annotated as corresponding to the homeodomain-like, Myb-containing protein. Its sequence is similar to that of the plant-specific GARP family of transcription factors (Hosoda et al., 2002). These proteins, including the Golden2-like proteins, play an important role throughout the plant’s life cycle (Ohama, Yanagisawa, 2024). In particular, among other processes, they control the development of chloroplasts and determine the quantitative aspects of photosynthesis (Chen M. et al., 2016). Our results suggest that this gene plays an active role in adaptation of CS wheat to soil drought. The genetic modification of the chromosome segment in the region of its localization led to a significant decrease in the stability of photosynthesis and shoot biomass in line 1021. Golden2-like (GLK) transcription factors have recently been considered as potential candidates for improving photosynthesis in agricultural crops (Hernández-Verdeja, Lundgren, 2024). Our data support the idea that GLK genes may be a promising biotechnological tool for improving drought tolerance in bread wheat, if the donor genotype is properly selected.

Three lines (1022, 1028 and 1034) had segments from Ae. tauschii in the region of marker Xgwm1419 in chromosome 2D. Additionally, line 1034 had introgression in the region of marker Xgwm157 (Fig. 1). According to GrainGenes data, genes functionally significant for drought tolerance are not localized in the region of this marker. This is probably why lines 1028 and 1034 were similar in terms of stability of the shoot biomass. At the same time, lines 1022 and 1028 differed from lines 1034 and 1021 in the response of stomatal apparatus to water deficit. We suggest that this phenomenon is associated with the gene TraesCS2D03G0081400 (2D:494675291–494678461 bp), localized relatively close to marker Xgwm1419. This gene encodes a protein, a member of the GTL1 family of transcription factors. GTL1 is known to be involved in the regulation of stomatal density, transpiration, stomatal conductance and, as a consequence, affects water use efficiency (Yoo et al., 2011). In addition, using RT-PCR, its significant expression was shown in many organs of wheat plants at the flowering stage, as well as an immediate (within 3 hours) response to osmotic stress (Zheng et al., 2016). The increase in transpiration and stomatal conductance in lines 1022 and 1028 and the decrease in WUE, especially in line 1022, could be associated with a change in the functionality of the GTL1 gene.

Lines 1021 and 1034 differed contrastingly for the stabi- lity of chlorophyll fluorescence indices (Fig. 3). In line 1021, unlike 1034, the real efficiency of PSII, the rate of electron transport, the index of functional activity of PSII (PIabs), and the integral index of functional activity of PSI and PSII (PItot) decreased under water deficiency. These differences are presumably due to introgression from Ae. tauschii into chromosome 2D. Line 1034 had the introgression in the region of marker Xgwm1419, which is located in coordinates 2D:472226450–472226470 bp, close to the TraesCS2D03G0058100 gene (coordinates 2D:480941598– 481111682 bp), encoding the PsbQ protein. The functions of this protein are associated with the coordination of the activities of the donor and acceptor functions of PSII and the stabilization of the active form of the light-harvesting complex of PSII (Ifuku et al., 2011). Introgression from Ae. tauschii in the region of marker Xgwm1419 could have a positive effect on the functioning of photosystem II, which makes the main contribution to chlorophyll fluorescence.

## Conclusion

A comparative study of the stability of photosynthesis and shoot biomass in wheat variety CS and secondary introgressive lines CS(Syin6x) for chromosome 2D showed significant diversity in these traits. Considering that the genotypes were grown under controlled conditions, the found differences in soil drought tolerance are presumably associated with introgressions from Ae. tauschii. Based on the results of the study, it can be concluded that the single introgression into the short arm of chromosome 2D limited by molecular markers Xgwm296 and Xgwm261 was favorable for drought tolerance. Introgression into the long arm of the same chromosome in the region of marker Xgwm1419 also supported drought tolerance. Introgression in this chromosome arm restricted by marker Xgwm539 was unfavorable for photosynthetic stability and shoot biomass. Markers Xgwm296 and Xgwm1419 can be recommended for the use in marker-assisted breeding of wheat for drought tolerance in cases where Ae. tauschii is used as a donor of genetic material

## Conflict of interest

The authors declare no conflict of interest.
